# Postures anomaly tracking and prediction learning model over crowd data analytics

**DOI:** 10.7717/peerj-cs.1355

**Published:** 2023-05-24

**Authors:** Hanan Aljuaid, Israr Akhter, Nawal Alsufyani, Mohammad Shorfuzzaman, Mohammed Alarfaj, Khaled Alnowaiser, Ahmad Jalal, Jeongmin Park

**Affiliations:** 1 Department of Computer Sciences, College of Computer and Information Sciences, Princess Nourah Bint Abdulrahman University, Riyadh, Saudi Arabia; 2 Department of Computer Science, Bahria University, Islamabad, Pakistan; 3 Department of Computer Science, Taif University, Taif, Saudi Arabia; 4 Department of Electrical Engineering, King Faisal University, Al-Ahsa, Saudi Arabia; 5 Department of Computer Engineering, Prince Sattam Bin Abdulaziz University, Al-Kharj, Saudi Arabia; 6 Department of Computer Science, Air University, Islamabad, Pakistan; 7 Department of Computer Engineering, Tech University of Korea, Sangidaehak-ro, Siheung-si, South Korea

**Keywords:** Anomaly detection, Compressive tracking Algorithm, Crowd based data, Data optimization, E-Learning, Fused dense optical flow, Fuzzy C mean, Gradient patches, Predication model, T-distributed stochastic neighbor embedding

## Abstract

Innovative technology and improvements in intelligent machinery, transportation facilities, emergency systems, and educational services define the modern era. It is difficult to comprehend the scenario, do crowd analysis, and observe persons. For e-learning-based multiobject tracking and predication framework for crowd data via multilayer perceptron, this article recommends an organized method that takes e-learning crowd-based type data as input, based on usual and abnormal actions and activities. After that, super pixel and fuzzy c mean, for features extraction, we used fused dense optical flow and gradient patches, and for multiobject tracking, we applied a compressive tracking algorithm and Taylor series predictive tracking approach. The next step is to find the mean, variance, speed, and frame occupancy utilized for trajectory extraction. To reduce data complexity and optimization, we applied T-distributed stochastic neighbor embedding (t-SNE). For predicting normal and abnormal action in e-learning-based crowd data, we used multilayer perceptron (MLP) to classify numerous classes. We used the three-crowd activity University of California San Diego, Department of Pediatrics (USCD-Ped), Shanghai tech, and Indian Institute of Technology Bombay (IITB) corridor datasets for experimental estimation based on human and nonhuman-based videos. We achieve a mean accuracy of 87.00%, USCD-Ped, Shanghai tech for 85.75%, and IITB corridor of 88.00% datasets.

## Introduction

Throughout human–computer contact, machine learning, user interface, intelligent observation, and crowd dynamics, the domain of human behavior has become a prominent subject of investigation. Between those domains, crowd dynamics has sufficient interest in digital recognition for a variety of problems, including density estimates (Chen et al., 2013), object tracking, surveillance, and crowded behavior identification (Akhter, 2020; Ghadi et al., 2022). Detecting crowd behavior involves detecting people’s psychological behaviors in a swarm context (Bera & Manocha, 2014). Through digital technologies (Rafique, Jalal & Kim, 2020a; Rafique, Jalal & Kim, 2020b), machine learning, pattern recognition, and object recognition methods, researchers provide the e-learning context for educational, public, and pedestrian statistics (Akhter, Jalal & Kim, 2021b; Gochoo et al., 2021; Alam et al., 2022). The rapid evolution of revised procedures and technologies for monitoring human activity leads to greater precision in the e-learning area (Adam et al., 2008). Intelligent technologies, especially realistic image-processing capabilities and ensemble learning, were also utilized in the field to understand the behavior of users *via* webcams (Mousavi et al., 2016).

A spatial connectivity examination was established to assess structural similarity throughout an image sequence. Using a variety of hypotheses to indicate spatially and temporally-associated associations across segmentation methods, researchers concluded a high percentage of success (Ryoo & Aggarwal, 2009). Each connection was characterized by redistributing the target region and determining local features using a learning algorithm (Akhter & Hafeez, 2022; Jalal, Akhtar & Kim, 2020; Ghadi et al., 2021; Akhter & Javeed, 2022). Due to this misunderstanding, they assumed a permanent environment and could not handle the natural world and e-learning approaches (Berlin & John, 2016). Researchers could identify inquisitive regions by considering the substantial swings in frequency components caused by locomotion. Researchers employed convolutional feature designations to characterize each focal point, but there were a few inaccuracies due to inaccurate recognition of essential locations and inter-actor image variances (Chattopadhyay & Das, 2016). They consider the Euclidean distance, angular velocity, interpersonal deceleration, hand positioning, foot orientation, and lower extremity surface, Zhan & Chang (2014) employed a visual organizational model to estimate the spot of adjacent vertebrae and establish the interconnections between them.

In the majority of existing human action data, Blank et al. (2005) human activities are captured in clean environments, and each visual often contains only a specific type of activity (*e.g.*, running or walking) performed by a single individual across the entire frame. Therefore, the foreground is often congested in actual surveillance scenarios, and video surveillance must identify the human movements of significance among a population (Azmat & Jalal, 2020; Javeed, Jalal & Kim, 2021; Ahmed, Jalal & Kim, 2019; Jalal, Khalid & Kim, 2020). In contrast to traditional activities like sprinting and jumping, researchers expect to discover whether visitors in a shopping complex want to take the goods off the market. Action identification in complicated contexts is significantly more challenging than in basic laboratory settings. It is difficult to correctly pinpoint human bodies in complex environments, such as those with dense backdrops or slightly blurred crowds (Akhter, Jalal & Kim, 2021b). In the absence of human engagement, cropping an object from a complicated image frequently results in severe distortion or infrequent wandering. There may also be misunderstandings in the wavelet transform.

A vast proportion of acts in the ordinary world are unique and brief. Although the human motion is continuous and the pace varies widely, it is difficult to determine the beginning or conclusion of these activities’ significance in real-life circumstances and the length of each one. Spatiotemporal domain anomalies are not detected in repetitive motions, including sprinting and sprinting, while they can significantly impact the recognition accuracy of non-repetitive operations, including reaching an object, snapping a photograph, and pressing an emergency button. Both these temporal and spatial inconsistencies significantly complicate the activity identification process (Ghadi et al., 2021). A primary method for overcoming these discrepancies seems to be to request proper labels from human laborers. The labelers must supply the region proposals of the entities as well as the starting and ending images of an intervention object. This task of labeling is exceedingly arduous. The recently established video that is several seconds long could take several months or decades. During the detection phase, researchers may potentially encounter difficulties with action alignment. Collecting well-aligned activity occurrences to enter into the classification model is challenging since the borders amongst continuous operations are typically hazy and the foreground is naturally chaotic.

Predicated on the above argument, current computer visual approaches developed to detect individualized behavioral responses are unsuitable for modeling and recognizing events in crowded settings. This has prompted the intelligence group to develop strategies for modeling and comprehending crowd behavior patterns. Recent research has focused extensively on modeling and detecting anomalous behaviors in multimedia data. Conventional studies that have been published differ fundamentally in regards to the kinds of aberrant behavior (*e.g.*, panic (Haque & Murshed, 2010), violent behavior (Hassner, Itcher & Kliper-Gross, 2012), and breakaway (Wu, Wong & Yu, 2013), categories of capabilities (density maps of low-level parameters, optical flow, directions, spatial and temporal attributes), modeling architectures and reinforcement learning (*e.g.*, Markov-like approaches, Bayesian approaches (Wang, Ma & Grimson, 2008)), and swarm.

For e-learning-based multiobject tracking and predication framework for crowd data *via* multilayer perceptron, this article recommends an organized method that takes e-learning crowd-based type data as input, based on usual and abnormal actions and activities. We perform some preprocessing for complex computational cost minimization and time-saving for prediction. After that, super pixel and fuzzy c mean are applied for more processing. For features extraction, we used fused dense optical flow and gradient patches, and for multiobject tracking, we applied a compressive tracking algorithm and Taylor series predictive tracking approach. The next step is to find the mean, variance, speed, and frame occupancy utilized for trajectory extraction. To reduce data complexity and optimization, we applied T-distributed stochastic neighbor embedding (t-SNE). For predicting normal and abnormal action in e-learning-based crowd data, we used multilayer perceptron (MLP) to classify numerous classes. We used the three crowd activity USCD-Ped, Shanghai tech, and IITB corridor datasets for experimental estimation based on human and nonhuman-based videos. All the dataset contains various view and various camera locations. The extensive research achievements of this article are as follows:

• E-learning-based method to predict pedestrian behavior in the crowd-based dataset.
• Multiple object tracking and human detection are performed *via* multilayer algorithms.
• Extraction sense organized features we extract various components, and to minimize the data replication, we utilized T-distributed stochastic neighbor embedding (t-SNE). For predicting normal and abnormal action in e-learning-based crowd data, we used a multilayer perceptron (MLP).

This article’s subcategories are as described in the following: We start by talking of related work, then introduce our platform technique, then describe the prototype model in depth, and conclude with a review of the research.

## Related Work

Utilizing spatial–temporal regions of interest described in creative and classification techniques is the most prevalent method for recognizing human actions (Beddiar et al., 2020). Numerous efforts have been made to augment spatial–temporal attention spots with essential information, such as hierarchical structures, indirect forms, local settings, 3D spin pictures and 3D cubes (Weng et al., 2018). Utilizing spatial–temporal key points simplifies the distinction between periodic movements, such as running and walking, as the synchronization difficulty in the video sequence is eliminated. Furthermore, spatial–temporal desire elements emphasize specific data rather than universal mobility, and if the camcorder is not static, identifying original geometric local features on living organisms in complicated settings may fall on crowded backdrops. Earlier approaches for human action recognition that are not dependent on spatial–temporal relevant features are limited to well-controlled contexts. Boiman & Irani (2007) described extracting highly collected video playback regions for recognizing irregular behaviors in basic background films. Rodriguez, Ahmed & Shah (2008) created a unique filter for analyzing the sorting behaviors of different activities. This method struggles to coordinate non-repetitive behaviors in difficult environments. However, some scholars attempted to mimic the human body’s architecture and evolution in the time domain. In Bobick (1997) demonstrate the problems of detecting actions as the variety of how motions are performed and how the correlation coefficient between person and environment emerges, confirming our spatial–temporal complexity.

Several researchers examined pedestrian behavior focused on social standards often observed in crowded public places (Zeng et al., 2014). The researcher analyzed the behaviors employed by pedestrians in such encounters and discovered the changes, such as pedestrians maintaining a specific range from one another, avoiding pedestrians approaching them, and pedestrians being capable of following the movement of other pedestrians in the area. Alahi et al. (2016) suggested a system that explicitly learned such connections by applying a socioeconomic structure to every individual’s Long Short-Term Memory (LSTM) structure.

Techniques to forecast the trajectories of pedestrians commenced with tracking because it is the appropriate second phase after detecting a person. Numerous research has estimated the location of pedestrians using the Kalman Filter (KF) and Particle Filter (PF) (Alahi et al., 2016). In Hariyono, Shahbaz & Jo (2015), as in related research work, the pedestrian position of a commuter is calculated on their location in two consecutive video frames concerning the vehicle’s location. Kataoka et al. (2015) deduced pedestrian purpose by identifying a walking engagement *via* pedestrian localization and examination. Schneider & Gavrila (2013) compare the Extended Kalman Filter (EKF) using single simulated data with the Interacting Multiple Model (IMM) techniques, which consolidates Constant Velocity (CV), Constant Acceleration (CA), and Constant Turning Rate (CR) (CT). In addition, researchers presented a dataset containing four aspects of pedestrian behaviour, namely walking, resting, curving in, and taking away, which was utilized extensively in the following research.

Keller & Gavrila (2013) devised a system for pedestrian path estimation and analyzed it with several methodologies, including GP, Probabilistic Hierarchical Trajectory Matching (PHTM), KF, and IMM at numerous time frames. When the specific positions of humans are known, the predicted performance was roughly comparable; however, GPDM and PHTM improved performance in pausing conditions. For reference purposes, the research also used human participants to anticipate whether pedestrians will halt or cross the roadway. Kooij, Schneider & Gavrila (2014) employed flipping dynamics (Linear Dynamical System (LDS)) for more precise path predictions. Researchers determined that certain future acts are more probable to take place based on past moves and current positions. Best & Fitch (2015) estimated the target location and ensuing trajectory. A region map was integrated into the model, and a Bayesian methodology was used to generate the confidence interval that captures the projected subsequent placement.

Multimedia and augmented reality strategies have proven to be valuable educational aids for decreasing pedestrian incidents in youngsters, reconditioning those with brain injuries, and learning essential driving skills. Simulators emphasize response, permit for gradated amounts of task sophistication, and enable the program to be adapted to each participant’s ability, offering programmed practical and individualized learning or training. Modules have recently been examined as instructional tools for senior students (Weiss, Naveh & Katz, 2003). The recommended simulator-based strategy includes panel discussions, realistic demonstrations, and two instruction sessions on precautionary behaviors and basic driving laws (Schwebel, Gaines & Severson, 2008). The purpose of the short-term simulation was to enhance the numerous driving strategies taught in classes and lectures. The benefits did not persist after 18 months (De Winter et al., 2007).

In contrast, a more dynamic and realistic style of emulation learning was utilized through practice, repetition, and individualized information. This method has been shown to improve the performance and capacities of older drivers, namely visual monitoring at intersections (Romoser & Fisher, 2009). More research is required to establish whether these improvements will persist. However, only a few studies have been conducted to determine whether a programmer combining behavioral and learning procedures in a safe and accurate traffic environment could assist older individuals in making better-stepping judgments (Roenker et al., 2003).

## Materials & Methods

**Figure 1:**
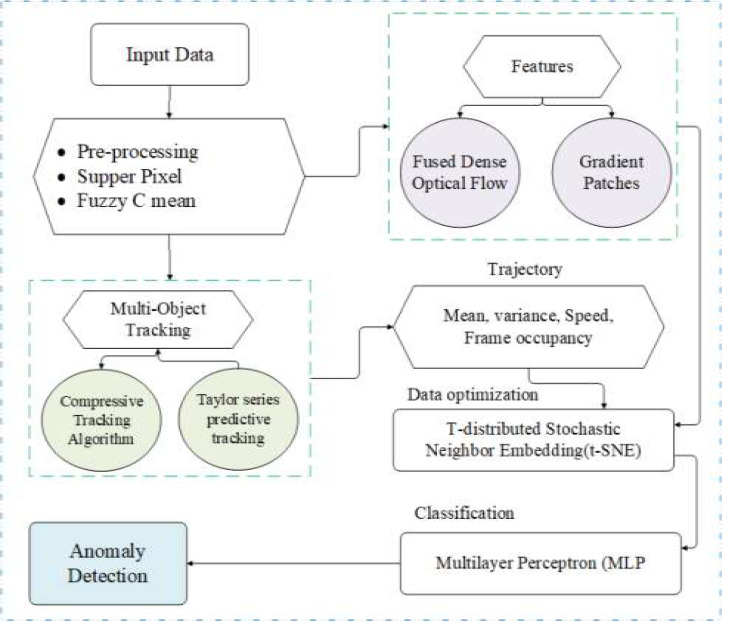
The proposed system design.

In this section, we discuss the main idea of our proposed methodology. Initially, we take RGB-based video data as input for our system. To reduce the computational cost, we applied some preprocessing steps such as noise reduction, frame conversion and RGB to binary conversion. After this, we apply the background subtraction method *via* the supper pixel approach and fuzzy C mean algorithm. The next step is tracking multi objects in input extraction features, namely fused dense optical flow and gradient patches. We have some trajectories: speed, mean–variance and frame occupancy. To minimize the extra features and save the cost of the system, we need an optimization approach for extracted data. For this, we apply t-SNE as a data optimizer and multilayer perceptron for normal and abnormal behaviour prediction. Figure 1 determines the proposed system design operational design.

Algorithm 1 provides a comprehensive picture of the suggested technique, including a description of the recommended strategy’s phases and a description of the technique’s main purposes, subfunctions, and formulas.

### Preprocessing of the data

While detecting human body features, several preparatory techniques are used to reduce computing time and expense. After converting media files to pictures, a motion blur filter is implemented to remove additional information. Fuzzy c mean and supper pixel-based separation were used for background removal and multiobject identification.

Fuzzy c-means with superpixels and similarity measure separation (Wu et al., 2013) standard Fuzzy c-means is a clustering technique that employs transfer learning and organizing variables by combining elimination assumptions and groupings facilities. Researchers enhanced it by utilizing superpixels as information because the FCM required less processing time than fuzzy c-means. Superpixels can be generated by implementing the Mahalanobis distance to the image features. In the FCM, researchers accomplished delineation using the hyperparameter *J*_MFCM_, a hyperparameter is a restriction whose significance is used to switch the learning process. which minimizes the proportional relationship of the complete sample points *X* containing superpixels, the clustering centers *pi*, and the memberships matrix *U*, whose are described as follows:

(1) \begin{eqnarray*}X= \left\{ {x}_{1},{x}_{2},\ldots \ldots .,{x}_{n} \right\} , P= \left\{ P,{P}_{2},\ldots \ldots .,{P}_{n} \right\} \end{eqnarray*}

(2) \begin{eqnarray*}U= \left[ {U}_{\mathrm{IJ}} \right] \in { \left[ 0,1 \right] }^{\mathrm{CXN}}\end{eqnarray*}

(3) \begin{eqnarray*}{J}_{\mathrm{MFCM}}=\sum _{J}^{N}\sum _{I}^{P}{u}_{ij}^{m}{d}_{M}^{2}.\end{eqnarray*}

Using

(4) \begin{eqnarray*}& & \forall \mathrm{j}\in 1,\ldots ,\mathrm{N},\mathrm{i}\in 1,\ldots ,\mathrm{c}:1\geq {\mathrm{u}}_{ij}\geq 0\end{eqnarray*}

(5) \begin{eqnarray*}& & \forall \mathrm{j}\in 1,\ldots ,\mathrm{N}:\sum _{i=1}^{c}{U}_{ij}, 1\lt \mathrm{m}\lt \mathrm{\infty }\end{eqnarray*}

where *N* is the statistics facts, *P* isthe whole quantity of modules, *u*_*ij*_ is the association gradation of fact *xi* in the *jth* collection, *m* is the weightiness that characterizes the gradation of woolliness and *dM* is the Mahalanobis distance among given data argument *xi* which is characterized as;

(6) \begin{eqnarray*}& & {d}_{m}=({x}_{i}-v)^{t}\sum -1({x}_{i}-v)^{t}\end{eqnarray*}

(7) \begin{eqnarray*}& & \sum = \frac{1}{n} \sum _{j=1}^{n}({x}_{i}-v)({x}_{i}-v)^{t}\end{eqnarray*}

where *V* demonstrates the mean vector for all illustrations. Algorithm 2 shows the detailed procedure of fuzzy c-means with Superpixels.

Fuzzy c-means (FCM) is a popular clustering algorithm that is often used for image segmentation. The algorithm uses a probabilistic approach to clustering, where each pixel in an image is assigned a membership value to each cluster rather than a challenging assignment to a single cluster. This allows for pixels to have partial membership in multiple clusters, resulting in a more accurate segmentation of an image. On the other hand, Superpixel-based segmentation is a technique that aims to segment an image into large, homogeneous regions (superpixels) rather than individual pixels. This can reduce image segmentation’s computational complexity and lead to more meaningful and coherent regions.

Combining FCM and superpixel-based segmentation for preprocessing can provide a powerful and efficient approach to image segmentation. FCM can segment the superpixels generated by the superpixel-based segmentation algorithm, resulting in a more accurate and computationally efficient image segmentation (Wu et al., 2013).

### Features extraction

In this subsection, we perform the extraction of features in which fused dense optical flow and gradient patches type features are extracted and mapped in the main features vector.

#### Fused dense optical flow

**Figure 2:**
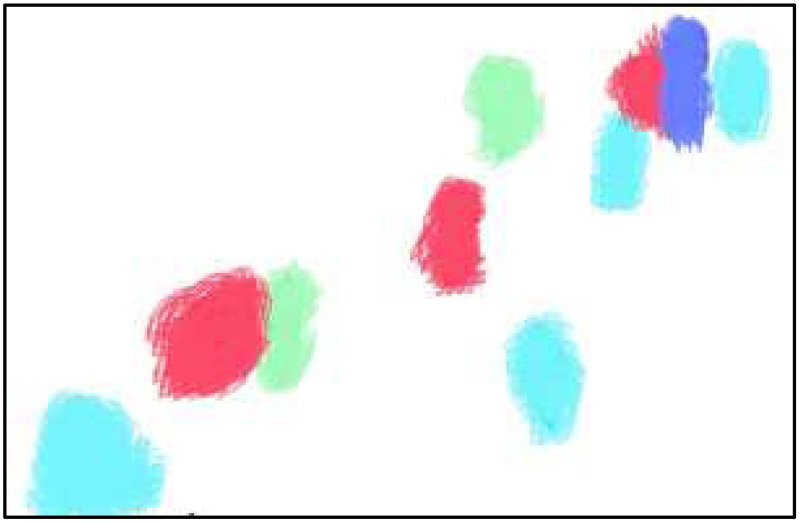
The visual representation of fused dense optical flow.

To obtain the fused dense optical flow patchwork, the continuous Lucas–Kanade methodology [40] is used to determine the horizontal and vertical fused dense optical flow ability at each frame, denoted as *fu* and *fv*. The flow intensity is therefore calculated using the Formula:

(8) \begin{eqnarray*}f=(fu)^{2}+(fv)^{2}\end{eqnarray*}

to generate a fused dense optical flow pattern as the kinematic attribute. Furthermore, the fused dense optical flow pattern is applied with a cross-patch framework to develop the optical flow patchwork. To achieve precision and reduce computational complexity, the recovered horizontal stripe and fused dense optical flow patterns are kept if at least 10 percent of their pixels are in motion and subsequently eliminated. Figure 2 shows the results of fused dense optical flow.

#### Gradient patches

To recognize simultaneous appearances and motion anomalies of image regions, we use the suggested multi-scale patchwork construction to identify their appropriate gradient patches as sources to correspondingly the presentation and mobility streams. By obtaining the gradient patches, we initially calculate the gradient intensity through each pixel in each video sequence using the approach described to generate the gradient pattern. Every gradient map has three components: the first and second pathways capture the readings in the image’s vertical and horizontal axes, which represent an item’s posture or structure accordingly. The middle stream includes the information from the video’s spatiotemporal component, representing the evolving picture over time. The cross-patch pattern is then used on the gradient images to generate local gradient patchwork.

The choice of feature representation depends on the task you want to perform and the characteristics of your data for Object detection. The Gradient patches can detect objects based on their shape and texture, while dense optical flow can detect objects based on their motion. Combining these features can improve object detection performance in images with static and moving objects. While gradient patches can be used for image classification to classify images based on their texture and shape, dense optical flow can be used to classify images based on their motion. Combining these features can improve image classification performance in images with static and moving objects (Fowlkes, Martin & Malik, 2003).

### Multiobject tracking

Detecting the items in every frame of a video is the preliminary stage in recognizing the presence of an aberration in crowded footage. The objects in the film suggest that the individuals in the image are engaged in various activities. That work employs footage captured in a congested location. The movie depicts numerous objects moving, walking, and bicycling, among other activities. One thing may overshadow another object in consecutive frames.

Consequently, the orientation of the elements in each frame must be identified to identify aberrant actions in a movie. Imagine the dense movie *A* containing *N* frames. To achieve the multiobject detection and tracking phase, we applied two robust algorithms: the compressive tracking algorithm and the Taylor series predictive tracking model. After getting both algorithms’ results, we optimized them and used them in further processing.

#### Object tracking based on the proposed Taylor series-based compressive approach

The second process in the AD method involves monitoring objects from one image to the next. This article uses a hybrid object tracking framework based on the predicted validation set employing the Taylor series (Yang, Zhang & Zhang, 2001) and the locational model employing a compressing technique (Zhang, Zhang & Yang, 2012). The predicted tracking based on the Fractional derivative uses the Taylor quadratic of the second degree to maximize monitoring precision. In the meantime, the compressing method can track the target while maintaining the picture composition of the subject.

#### Tracking model based on the proposed Taylor series-based compressive approach

The recommended TSC technique offers the completed object tracking function. The suggested TSC method relies on the monitoring location value derived using the TSP framework and CT methodology. Equation (9) represents the particle monitoring equation for the recommended TSC method. The suggested monitoring model provides the precise location of an object within the image sequence.

(9) \begin{eqnarray*}B= \frac{{B}_{T}+{B}_{C}}{2} \end{eqnarray*}

where, the *B* denotes the position of an object in the given frame. The variable *B*_*T*_ and *B*_*c*_ are the tracked outcomes of Taylor series and compressive methods.

Compressive tracking is a method that uses sparse representations to track objects in images. The algorithm starts by selecting a set of basic functions and then represents the object in the image as a sparse linear combination of these basis functions. This allows the algorithm to track the object by updating the sparse coefficients of the object in each frame. Compressive tracking is robust to object deformations, partial occlusions, and cluttered backgrounds (Ballesteros-Pérez, Elamrousy & González-Cruz, 2019).

The Taylor series predictive tracking approach, on the other hand, is a method that uses a Taylor series expansion to model the motion of an object in images. The algorithm starts by assuming that the object’s motion can be approximated by a Taylor series expansion around the object’s current position. The algorithm then uses this model to predict the object’s place in the next frame and update the object’s position accordingly. This approach is efficient and robust to small changes in the object’s motion (Luo et al., 2022).

Both algorithms are suitable for object detection in images, as they are robust to object deformations and occlusions and can handle cluttered backgrounds. The choice of algorithm depends on the specific problem and the characteristics of the data. For example, if the object’s motion is known to be smooth, the Taylor series predictive tracking approach may be more suitable. On the other hand, if the object’s motion is known to be non-smooth, the compressive tracking algorithm may be more appropriate. Combining both algorithms achieves more accurate and optimized results (Luo et al., 2022).

### Trajectory

In this section, the extraction of trajectory values from given frames in which we implemented the mean, variance, speed and frame occupancy trajectory extraction technique pulls the essential components from the objects monitored by the measurement model. This research retrieves data including mean, variance, speed, and frame occupancy from monitored objects. The retrieved features comprise the feature map. Following is an explanation of the trajectory involved in the trajectory extraction procedure.

#### Mean

The object’s location varies from image to image in a stream, and the range between the object’s positions in each image is estimated. The average area covered by each object among consecutive sets is then calculated and designated a trajectory. The average distance measured from image to image is represented as follows:

(10) \begin{eqnarray*}P \left( {A}_{o},{A}_{o+1} \right) = \frac{\sum _{o=1}^{N}C}{N} .\end{eqnarray*}

Where the *C* denotes the distance trekked by the entity among the *o*th and the (*o* + 1)th frame. The term *N* denotes the total amount of structures.

#### Variance

Likewise, the variance of the measurement point is determined for the entity for each image sequence. The conflict between the frames to frames traveled by the item is described as follows:

(11) \begin{eqnarray*}\vartheta \left( {A}_{o},{A}_{o+1} \right) = \frac{\sum _{o=1}^{N}(C-P)^{2}}{N} \end{eqnarray*}

where *ρ* is the mean values

#### Speed

The speed is the next component of the trajectory extraction procedure. Several items in the footage flow at various rates amongst each image. The pace allows the analyst to assess the object’s functioning. The ratio of the location to the moment is determined by the pace of the object’s position. The speed is expressed in the mathematical model by Eq. (12).

(12) \begin{eqnarray*}\mathrm{Speed}= \frac{C}{\delta } .\end{eqnarray*}

Where, the *C* expresses the distance stimulated by the entity from one frame to the next available frame, and the term *δ* denotes the time consumed by the object to complete the distance *C*.

#### Frame occupancy

The frame occupancy attribute specifies the space an entity occupies for each frame. Whenever objects move from one image to the next, the region dominated by an entity in each frame varies. By calculating the area, the structure of the thing may be determined, allowing for its easy recognition. Consequently, it is required to identify the space affected by each element within the frame.

(13) \begin{eqnarray*}& & {F}^{o}=\sum _{b=1}^{K}\sum _{v=1}^{L}{J}_{bv}\end{eqnarray*}

(14) \begin{eqnarray*}& & {J}_{bv}= \left\{ \begin{array}{@{}l@{}} \displaystyle 1;\text{if object} \\ \displaystyle 0;\text{otherwise}. \end{array} \right. \end{eqnarray*}

(Algorithm 3) A detailed overview of the Gradient Patches and Fused Dense optical flow features extraction methodology is shown.

### Data optimization and prediction

After mapping all the features and trajectories in the main features vector, we must apply some functions and algorithms to the optimized features vector. For this, we apply t-SNE based data optimization approach, which provides us with an optimized features vector. We used this vector in our next process, prediction and classification. There are vital techniques to accomplish this: retaining the attributes with the instruments varies and removing irrelevant details, or changing the existing feature set into a reduced collection of new features with roughly the same fluctuation as the initial edition. The t-distributed Stochastic Neighbor Embedding (t-SNE) algorithm (Der Maaten & Hinton, 2008) used in this study is a non-linear features extraction procedure that turns all categories with differing feature values into optimal additional columns. As its name suggests, this strategy is based on stochastic distribution and is solely concerned with preserving the variance of surrounding values. During the trials conducted by the researcher, the frequency of neighboring points, commonly known as complexity, was adjusted to *h* and the number of repetitions was changed to *t*.

The t-SNE is an effective method that maintains the model’s local and international representation. In other phrases, the calculated low-dimensional map includes the same amount of important structure as the original high-dimensional material upon feature reduction by t-SNE. For the t-SNE method to function, a conditional probability across combinations of high-dimensional elements must be constructed. The likelihood of identical items is considerable, while the possibility of divergent locations is minimal.

Underneath this distribution function, the concentration of all vertices *x*_*j*_ is collected and renormalized for all vertices. This concludes that each location has its probability in a sequence of values (*P*_*ij*_) for all endpoints, which can be described using formula.

(15) \begin{eqnarray*}{p}_{j{|}i}= \frac{\exp \nolimits (-{|}{|}{x}_{i}-{x}_{j}{|}{{|}}^{2}/2{\sigma }_{i}^{2})}{\sum \exp \nolimits (-{|}{|}{x}_{i}-{x}_{k}{|}{{|}}^{2}/2{\sigma }_{i}^{2})} .\end{eqnarray*}

The subsequent step is to create a comparable likelihood function over the weak graph’s components. In place of a random variable, an independent sample *t*, including one level of flexibility, often defined as the symmetric allocation, is employed here. This leads to a second iteration of probabilities *Q*_*ij*_ in low-dimensional reality, which can be defined using Eq. (15).

(16) \begin{eqnarray*}{q}_{ij}= \frac{(1+{|}{|}{y}_{i}-{y}_{j}{|}{{|}}^{2})^{-1}}{\sum (1+{|}{|}{y}_{k}-{y}_{j}{|}{{|}}^{2})^{-1}} .\end{eqnarray*}

After acquiring the two sets of likelihood, their distribution is estimated using Kullback-Liebler dive deviation (*KL*), as demonstrated by Eq. (16). If the *KL* dispersion value is small, it indicates that the two populations are similar. If the probabilities are equal, then the *KL* diverging measurement will be 0.

(17) \begin{eqnarray*}KL \left( P{|}{|}Q=\sum {p}_{ij}\mathit{log} \frac{{p}_{ij}}{{q}_{ij}} \right) .\end{eqnarray*}

Finally, gradient descent is utilized to minimize the KL objective functions. A t-SNE matrix reflecting the relationships between the potential contributors is constructed during optimization. Algorithm 4 shows the detailed overview of the t-SNE-based data optimization approach.

The main reason behind using t-SNE for data optimization is that it can preserve the local structure of the data. This means that similar data points in the high-dimensional space will be mapped close to each other in the low-dimensional space. This can be useful for image feature data because it can help to preserve the relationships between similar features in the data.

Additionally, t-SNE can reveal patterns and structures in the data that may not be obvious in the high-dimensional space. This can be particularly useful for image feature data, as it can help identify patterns and features necessary for a specific task or application (Linderman & Steinerberger, 2022).

### Prediction and classification

The extracted optimum features vector is used as input of MLP, an MLP architecture consists of numerous layers of feed-forward-connected perceptrons. As a classification model, the return of the protective layer is finished using a smooth procedure. As the current execution of MLP remains restricted to the validation process, designers chose normal optimum over soft-max to optimize FPGA capacity requirements (Gaikwad et al., 2019). The suggested MLP architecture comprises a source, production, and a hidden layer. Layer 1 is the input data that specifies various *I* characteristics. Typically, MLP consists of one maybe more hidden layers, and a hidden layer is used to minimize classification delay and computing resource consumption. A hidden layer (Layer 2) of the full MLP statistical model comprises *j* covert activation functions. The invisible gradient production framework is presented in (2).

(18) \begin{eqnarray*}{m}^{2}={a}_{1} \left\vert {g}^{2}\times {m}^{1}\times {B}^{2} \right\vert .\end{eqnarray*}

Where *g*^2^ is the weight matrix of MLP *m*^1^ is the output matrix while *B*^2^ is the bias function of MLP. The MLP sorts input characteristics into “ *k*” categories. Consequently, the feature extractor (Layer 3) is composed of “ *k*” emission autoencoder. The production matrix of this activation function is computed using the following formula: (3)

(19) \begin{eqnarray*}Y={a}_{2} \left\vert {g}^{3}\times {m}^{2+}{B}^{3} \right\vert .\end{eqnarray*}

**Figure 3:**
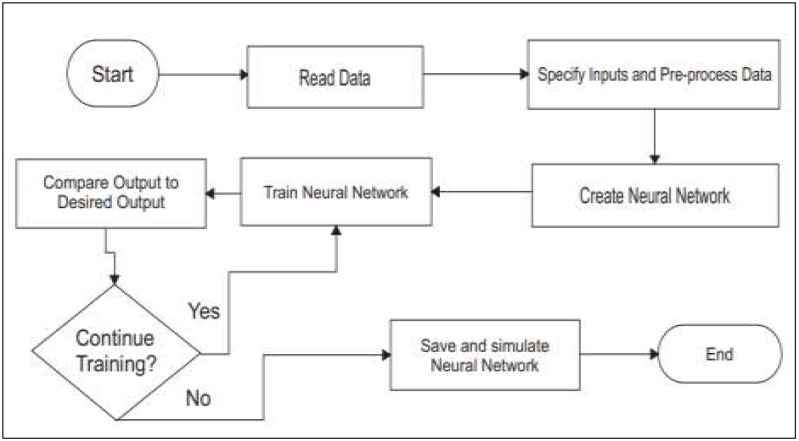
Flow chart for multilayer perceptron.

**Figure 4:**
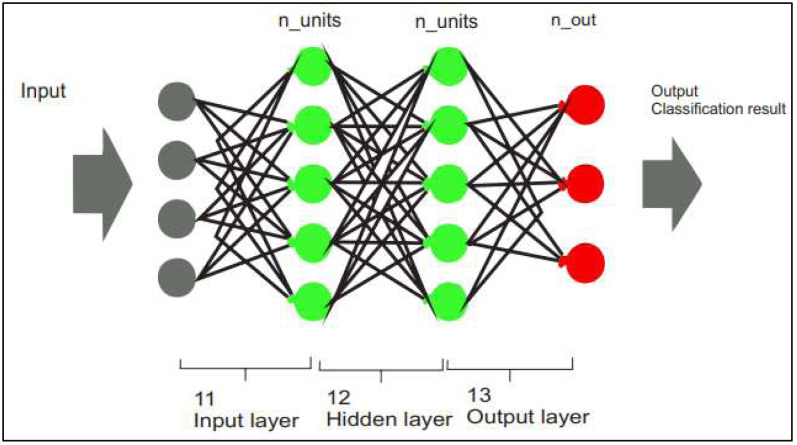
Multilayer perceptron model overview.

Where *g*^3^ is the weight matrix of MLP *m*^2^ is the output matrix while *B*^3^ is the bias function of MLP. The figure shows the detailed model of the multilayer perceptron. Figure 3 shows the flow chart of the multilayer perceptron, and Fig. 4 shows the multilayer perceptron model overview.

The selection criterion for using a Multilayer Perceptron (MLP) classifier for object detection in image-based data depends on the specific task and characteristics of the data. While we considered the complexity of the problem: MLP classifiers can model non-linear decision boundaries, which can be useful for object detection problems where the objects have complex shapes or are in non-uniform backgrounds. Additionally, we have used the 2nd criterion, the number of classes: MLP classifiers can handle many classes, which can be useful for object detection problems where there are many different object classes to be detected (Gaikwad et al., 2019).

We have used the grid search when tuning hyperparameters for a multilayer perceptron (MLP) applied to image data, which is a simple and effective method that can be used to find the optimal combination of hyperparameters. It works well for image data because it allows you to specify a range of values for each hyperparameter and test all possible combinations (Gaikwad et al., 2019).

## Results

### Dataset descriptions

The UCSD Anomaly Detection Dataset was collected using a static webcam at a height above pedestrian paths. The total population in the pathways ranged from minimal to highly dense. In a typical environment, the video depicts pedestrians. Either causes abnormal occurrences: distribution of non-pedestrian objects on walkways results in atypical pedestrian movements. Bicyclists, skateboarders, tiny carts, and pedestrians traversing a path or the adjacent grass often appear as anomalies. A few wheelchair-using individuals were also documented. All irregularities are commonly occurring; people were not manufactured for data collection. The data was divided into two subgroups, each corresponding to a particular scene. Each scene’s prerecorded material was split into clips containing approximately 200 images. Peds1: footage showing groups of individuals walking towards and distant from the webcam, with some horizon displacement. There are 34 demonstration video segments and 36 short testing videos included.

The Shanghaitech dataset is a despite the common for counting crowds. It contains 1198 categorized photographs of groups. Part-A of the collection has 482 photos, whereas Part B includes 716 images. Train and test selections for Part-A comprise 300 and 182 pictures, correspondingly. Part B is divided into training and evaluation subsets of 400 and 316 illustrations, respectively. Each individual in a crowd photograph is marked with a point at the center of the head. The collection contains a total of 330,165 categorized individuals. Images for Part-A were gathered on the world wide web, whereas images for Part B were gathered on the major thoroughfares of Shanghai.

To standardize classification methods for the activity of irregular action detection, researchers introduce a dataset consisting of community activities, including the opposition, chasing, fighting, and sudden running, as well as single individual operations, including attempting to hide the face, disturbing the peace, unoccupied personal belongings, transporting a questionable component and cycling (in a pedestrian area). Researchers anticipate that such a collection will inspire human behavior analysis studies considering individual or many human interactions. It is designated as the IITB-Corridor dataset. IITB-Corridor is a large-scale surveillance dataset that will be publicly accessible for free research.

### Experiment I: the human detection accuracies

Table 1 represents the outcomes of actual human detection and recognition over the UCSD dataset. Column 1 shows the sequence number of frames, *C*—2 for the actual human track and *C*—3 for the successful detection of humans, *C*—4 for the failure rate and *C*—5 for the accuracy. We achieve mean accuracy for the UCSD dataset is 90.34%.

**Table 1:** Actual human detection and identification accuracy over UCSD dataset.

Sequence No	Actual Track	Successful	Failure	Accuracy
5	5	5	0	100.0
10	5	5	0	100.0
15	5	5	0	100.0
20	6	5	1	83.33
25	6	5	1	83.33
30	6	6	0	100.0
35	7	6	1	85.71
40	7	6	1	85.71
45	8	6	2	75.00
**Mean accuracy = 90.34%**				

Table 2 represents actual human detection and recognition outcomes over the Shanghai tech dataset. Column 1 shows the sequence number of frames, *C*—2 for the actual human track and *C*—3 for the successful detection of humans, *C*—4 for the failure rate and *C*—5 for the accuracy. We achieve mean accuracy for the Shanghai tech dataset is 91.92%.

**Table 2:** Actual human detection and identification accuracy over Shanghai tech dataset.

Sequence No	Actual Track	Successful	Failure	Accuracy
5	6	6	0	100.0
10	6	6	0	100.0
15	6	6	0	100.0
20	7	7	0	100.0
25	7	6	1	85.71
30	8	7	1	87.50
35	8	7	1	87.50
40	9	7	2	77.77
45	9	8	1	88.88
**Mean accuracy = 91.92%**				

Table 3 represents actual human detection and recognition outcomes over the IITB corridor dataset. Column 1 shows the sequence number of frames, *C*—2 for the actual human track and *C*—3 for the successful detection of humans, *C*—4 for the failure rate and *C*—5 for the accuracy. We achieve mean accuracy for the IITB corridor dataset is 89.87%.

**Table 3:** Actual human detection and identification accuracy over IITB Corridor dataset.

Sequence No	Actual Track	Successful	Failure	Accuracy
5	5	5	0	100.0
10	5	5	0	100.0
15	5	5	0	100.0
20	6	5	1	83.33
25	6	5	1	83.33
30	6	5	1	83.33
35	7	6	1	85.71
40	7	6	1	85.71
45	8	7	1	87.50
**Mean accuracy = 89.87%**				

### Experiment II: behavior prediction accuracy

We used multilayer perceptron to predict human normal and abnormal action, which provides robust accuracy over crowd-based data and e-learning environments. The Leave One Subject Out (LOSO) cross-validation technique estimates the design technique. In Table 4, the confusion matrix representation over the UCSD dataset shows the mean accuracy rate and error rate. In Table 5, the confusion matrix representation over the Shanghai tech dataset shows the mean accuracy rate and error rate. In Table 6, the confusion matrix representation over the IITB corridor dataset shows the mean accuracy rate and error rate.

**Table 4:** Confusion matrix of proposed E-learning method over UCSD dataset.

Scene No	Anomaly detection	Error rate
Scene 01	85.00	15.00
Scene 02	89.00	11.00
Mean accuracy	87.00%	13.00%

**Table 5:** Confusion matrix of proposed e-learning method over Shanghai tech dataset.

Scene No	Anomaly detection	Error rate
Scene 01	84.00	16.00
Scene 02	87.50	12.50
Mean accuracy	85.75%	14.25%

**Table 6:** Confusion matrix of proposed e-learning method over IITB Corridor dataset.

Scene No	Anomaly detection	Error rate
Scene 01	87.50	12.50
Scene 02	88.50	11.50
Mean accuracy	88.00%	12.00%

### Experiment III: comparison with other classification algorithms

**Figure 5:**
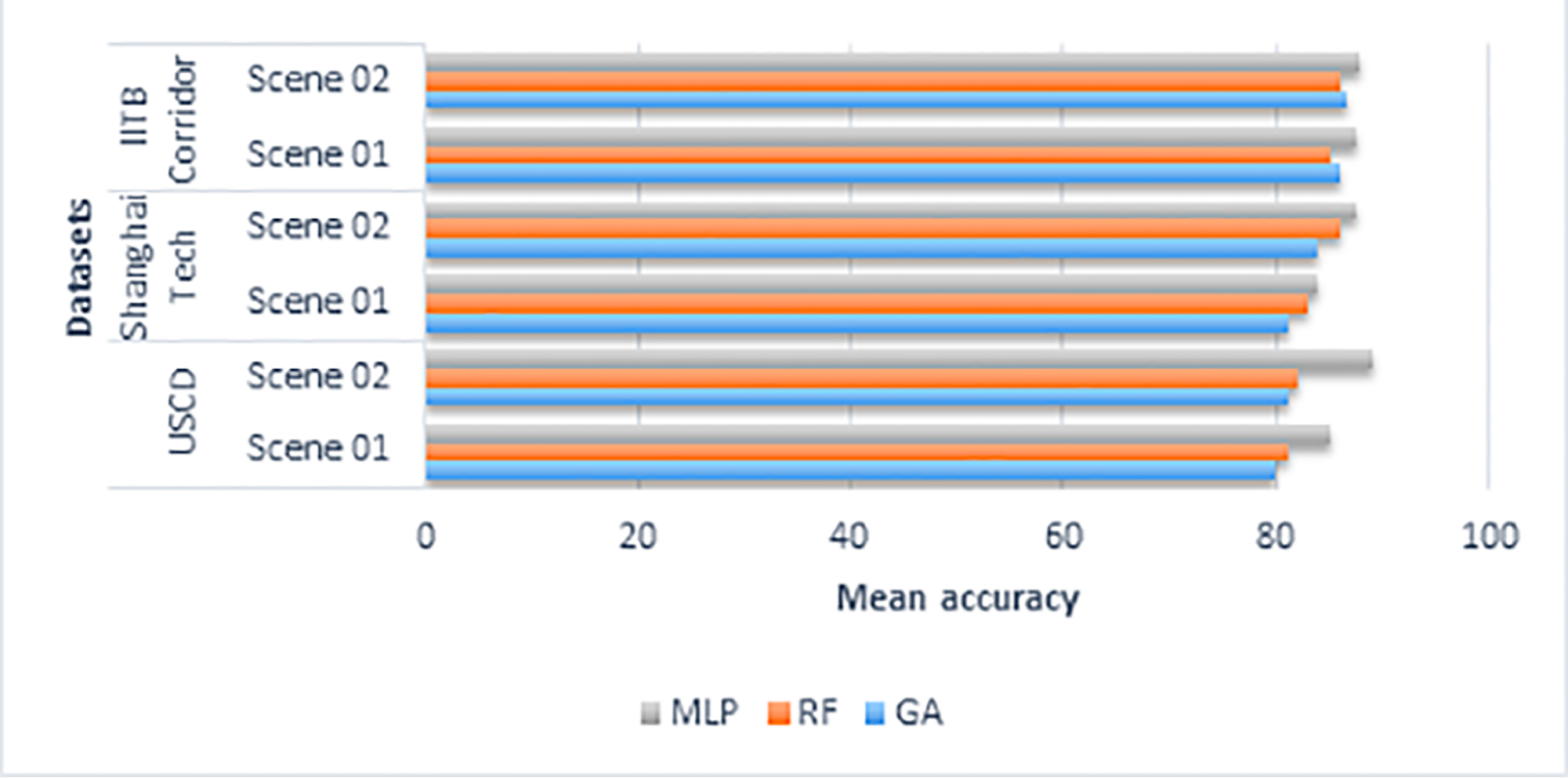
The detailed results of the comparison of other classifications algorithms with multilayer perceptron in bar chart format.

To evaluate our proposed method, we compare it with other machine learning classification algorithms, namely genetic algorithm and random forest. Fig. 5 shows the detailed results of the comparison in bar chart format.

### Experiment IV: comparison with other approaches

This section discusses the detailed comparison with other methods to evaluate our proposed method. Table 7, shows the detailed results. While Table 8, shows the comparison with other classification methods.

**Table 7:** Comparison with other approaches.

	USCD	Shanghai Tech	IITB corridor
ConvAE (Hasan et al., 2016)	81.00%	–	–
MDT (Jain & Bansal, 2021)	81.80%	–	–
Predication Net (Liu et al., 2018)	–	72.80%	–
BMAN (Lee, Kim & Ro, 2019)	–	76.20%	–
MTTP (Rodrigues et al., 2020)	–	–	67.12#
Morais (Morais et al., 2019)	–	–	64.27%
VABD (Li et al., 2021)	81.14%	78.20%	72.24%
Proposed Method	87.00%	85.75%	88.00%

**Table 8:** Comparison of other classification approaches with Multilayer perceptron.

**IITB Corridor dataset**					
Scene No	SVM	KNN	LDA	naive Bayes	Multilayer perceptron
Scene 01	80.00	81.50	80.50	79.50	87.50
Scene 02	78.00	83.50	82.50	77.50	88.50
Mean accuracy	79.00%	82.50%	81.50%	78.50%	88.00%
**Shanghai tech dataset**					
Scene No	SVM	KNN	LDA	naive Bayes	Multilayer perceptron
Scene 01	79.00	77.50	81.50	80.50	84.00
Scene 02	77.30	81.30	83.00	81.50	87.50
Mean accuracy	78.15	79.40	82.25	81.00	85.75%
**UCSD dataset**					
Scene No	SVM	KNN	LDA	naive Bayes	Multilayer perceptron
Scene 01	80.00	81.50	80.50	79.50	85.00
Scene 02	78.00	83.50	82.50	77.50	89.00
Mean accuracy	79.00%	82.50%	81.50%	78.50%	87.00%

## Scope of the Article

This suggested methodology is based on static and dynamic video-based data, which can be real-time or stored. This system provides complete accuracy of normal and abnormal action, behaviour and event detection. We can apply this system to surveillance systems, airport monitoring systems, traffic monitoring, law enforcement, medical system, intelligent home management, and educational and emergency system. While we have some limitations in this article, complex background, night effects, and high-frequency light effects may create an issue in finding complete information and a high error rate.

## Conclusion

This research article uses video-based data to predict human behavior and normal and abnormal events and classify the prediction results. The system can work on real-time data, surveillance cameras, or recorded video data. Initially, we start from the video as input of the system and perform some preprocessing steps, noise reduction, fuzzy c mean, and supper pixel the some of the preprocessing steps. The next step is to track multiobject from extracted data. We applied the compressive tracking algorithm and the Taylor series predictive tracking approach. We used robust and sense-aware features such as fused dense optical flow and gradient patches for the features extraction framework. The next step is to find the mean, variance, speed, and frame occupancy utilized for trajectory extraction. To reduce data complexity and increase the optimization of extracted data, we applied T-distributed stochastic neighbor embedding (t-SNE). For predicting normal and abnormal action in e-learning-based crowd data, we used multilayer perceptron (MLP) to classify numerous classes. We used the three crowd activity USCD-Ped, Shanghai tech, and IITB corridor datasets for experimental estimation based on human and nonhuman-based videos. We achieve a mean accuracy of 87.00%, USCD-Ped, Shanghai tech for 85.75%, and IITB corridor for 88.00% of datasets. At the same time, the mean accuracy of human detection achieved 90.34%, USCD-Ped, Shanghai tech for 91.92%, and IITB corridor for 89.87% of datasets. We compare the proposed method with other state-of-the-art methods, showing our system’s significant improvements. We find features such as 2D and 3D mesh, skeleton, body parts movement and texton-based segmentation over various complex datasets for future directions.

##  Supplemental Information

10.7717/peerj-cs.1355/supp-1 Supplemental Information 1 MDF Optical Flow Analysis Pipeline: A MATLAB Script for Processing and Analyzing Motion Direction Flow Data

10.7717/peerj-cs.1355/supp-2 Supplemental Information 2 Data Preprocessing Toolkit: An Open-Source Library for Cleaning, Transforming, and Optimizing Data for Machine Learning

10.7717/peerj-cs.1355/supp-3 Supplemental Information 3 IITB corridor dataset

10.7717/peerj-cs.1355/supp-4 Supplemental Information 4 ShanghaiTech

10.7717/peerj-cs.1355/supp-5 Supplemental Information 5 UCSD Anomaly Detection

## Additional Information and Declarations

### Data Availability

The following information was supplied regarding data availability: The raw measurements are available in the Supplemental Files.
